# Beta-defensin genomic copy number is not a modifier locus for cystic fibrosis

**DOI:** 10.1186/1477-5751-4-9

**Published:** 2005-12-07

**Authors:** Edward J Hollox, Jane Davies, Uta Griesenbach, Juliana Burgess, Eric WFW Alton, John AL Armour

**Affiliations:** 1 Institute of Genetics, University of Nottingham, Nottingham, UK; 2 Department of Gene Therapy, Faculty of Medicine at the National Heart and Lung Institute, Imperial College, London, UK; 3 Paediatric Respiratory Disease, Royal Brompton Hospital, London, UK; 4 Adult Cystic Fibrosis, Royal Brompton Hospital, London, UK

## Abstract

Human beta-defensin 2 (DEFB4, also known as DEFB2 or hBD-2) is a salt-sensitive antimicrobial protein that is expressed in lung epithelia. Previous work has shown that it is encoded in a cluster of beta-defensin genes at 8p23.1, which varies in copy number between 2 and 12 in different individuals. We determined the copy number of this locus in 355 patients with cystic fibrosis (CF), and tested for correlation between beta-defensin cluster genomic copy number and lung disease associated with CF. No significant association was found.

## Background

Cystic fibrosis (CF) is an autosomal recessive genetic disease in epithelia (Online Mendelian Inheritance in Man (OMIM) 219700, ), with chronic lung infection being the major cause of morbidity and mortality. It has a a carrier frequency of about 1 in 25 in the United Kingdom, and the disease is due to mutations in the cystic fibrosis transmembrane conductance regulator gene (CFTR) gene, which encodes a membrane chloride channel (OMIM 602421). There is a wide spectrum of different mutations known to cause CF, but the most common is the ΔF508 mutation, which exists in the European population as a polymorphic allele with a frequency of between 2 and 3%.

The course of lung infection can vary greatly between patients with the same CFTR mutation, and despite the wide spectrum of disease causing mutations, only a few correlate with lung disease severity. The search for genetic loci modifying lung disease in CF patients has produced several candidates, with the gene for mannose binding lectin (MBL) being the most notable. MBL is a part of the innate immune system, and low MBL in serum has been associated with infections in both adults and children [1,2]. Several studies have reported links with severity of CF lung disease, although the conclusion regarding the impact of the heterozygous deficient state differ amongst these [3-5].

Like MBL, beta-defensins are an important part of innate immunity. The peptide human beta-defensin 2 (encoded by the gene *DEFB4*, also known as DEFB2 or hBD-2) has been identified as a salt-sensitive antibacterial agent expressed in the lung airway [6]. Given the chronic bacterial colonisation of the lungs, this is a potential candidate for a CF modifier locus. It is possible that variation in DEFB4 levels could cause changes in the antibacterial effectiveness of airway surface fluid. Deterioration in lung function during clinical progress of CF is correlated with *DEFB4 *expression levels, suggesting again that DEFB4 has an important antibacterial role in airway surface fluid [7]. Additionally, *DEFB4 *expression is up-regulated by mucoid *Pseudomonas aeruginosa*, and has very effective bactericidal activity against it [8,9], but another important pathogen in CF lungs, *Burkholderia cepacia*, is not killed by airway surface fluid expressing *DEFB4 *[10].

Three beta-defensins are expressed in significant levels in airway epithelia: human beta-defensin 1 (*DEFB1*) which is expressed in the lung [11], human beta-defensin 3 (*DEFB103*, also known as DEFB3 or hBD-3) which is expressed in the trachea [12], and *DEFB4*, also expressed in the lung [6]. All three genes are part of a beta-defensin cluster at chromosomal locus 8p23.1 [13]. This cluster of beta defensins, with the exception of DEFB1, has been shown to be on a repeat unit of at least 260 kb which varies in copy number in different people. Individuals can have between 2 and 12 copies, with most individuals having 3, 4 or 5 copies, and copy number is correlated with basal unstimulated expression level of *DEFB4 *in lymphoblastoid cell lines [14]. There have been several genome-wide studies confirming this copy number variation [15-17], and they highlight this type of large scale genomic polymorphism as an underappreciated source of phenotypic variation. Indeed, there is evidence that variable *CCL3L1 *gene copy number is associated with susceptibility to HIV infection [18].

The hypothesis that we wished to test in this study was that copy number variation of this beta-defensin cluster (and, by implication, *DEFB4*) is associated with lung disease in CF patients.

## Results

**Figure 1:**
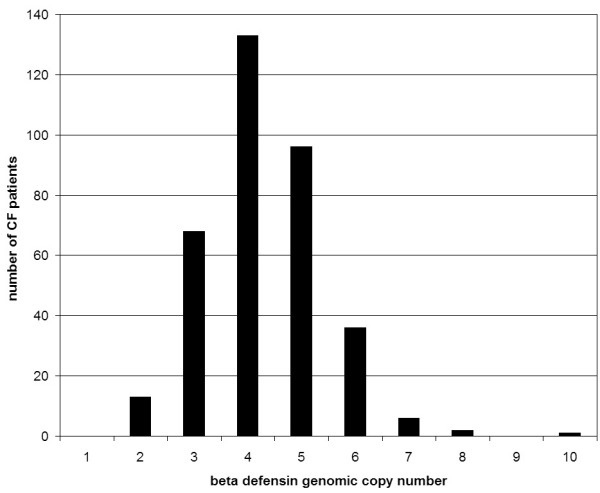
Beta-defensin diploid copy number distribution in the cohort of 355 cystic fibrosis patients analysed.

We determined beta-defensin cluster copy number for 355 CF patients, of whom 343 had full clinical data. 159 patients were ΔF508 CFTR homozygotes, 141 were ΔF508 CFTR heterozygotes and 43 had non-ΔF508 mutations on both chromosomes. The mean age was 29.1 ± 8.9 years and 57% were male. Figure 1 shows the distribution of beta-defensin copy number phenotypes in the full CF patient cohort. There was no difference in the copy number phenotype distribution between this cohort and a random sample of 167 normal UK individuals (E.J.Hollox and J.A.L.Armour unpublished data).

Each analysis was performed with the whole cohort, and with the ΔF508 CFTR homozygotes only. There were no significant correlations between beta-defensin copy number and each respiratory clinical parameter measured (mean and current FEV_1_, mean and current FVC). The patients were then arbitrarily divided into two groups based on copy number: one group comprising patients with 3 and fewer copies of the β-defensin region (n = 77), and another group with patients who have 4 copies and more (n = 266). Differences in lung function and inflammatory marker status were tested using two-tailed Mann-Whitney U-tests, differences in infection status were tested using χ^2 ^tests. With this sample size, a difference of 9% in FEV_1 _median between the two groups could be detected with 80% power. No significant differences between any two groups were seen.

**Figure 2:**
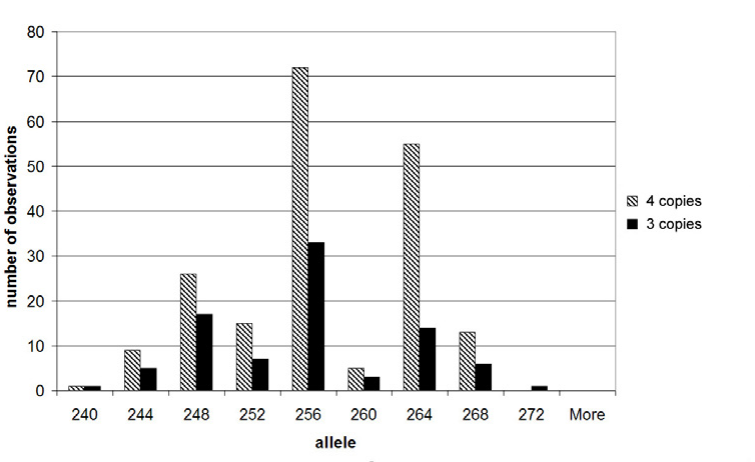
Allelic distribution of DEFB4 microsatellite (EPEV-2) alleles in individuals with three and four copies of the beta-defensin gene cluster.

We also decided to determine the effectiveness of microsatellite dosage ratios in reinforcing, or possibly acting as a replacement for, MAPH in determining copy number. We analysed a subset of samples with copy numbers of three or four by determining the genotype of a microsatellite in the intron of *DEFB4*. The allelic distributions are shown in Figure 2. Out of 114 samples tested, 81 gave informative results, of which 71 (88%) agreed with the determination by MAPH. The samples that differed typically gave unrounded MAPH scores between the two values (range 3.42–3.54 or 4.5–4.54), and may be due either to error rate or, perhaps more likely given the small confidence intervals of each MAPH result (see Methods) and its reliability for other loci [19-23], genuine copy number heterogeneity between probes across the repeats. We should be able to distinguish between genuine copy number heterogeneity and MAPH error rate by analysing the copy number estimate given by the DEFB4 probe alone, which is 735 bp from the microsatellite in the intron of *DEFB4*, and should report the same copy number as the microsatellite. Analysis of this copy number estimate for the 10 discrepant results shows that it agrees with the microsatellite estimate for 5 samples. Therefore, the error rate in distinguishing 3 from 4 copies by MAPH is 5/81 = 6%. The result also suggests that copy number heterogeneity involving the DEFB4 probe (probe F) has a frequency of around 6%. In addition, copy number heterogeneity involving MAPH probe H has been found in several well-characterised individual DNAs (E.J.Hollox, J.A.L.Armour and J.C.K.Barber, unpublished data).

## Discussion

This is the first study to test the hypothesis, raised in a previous paper [14], that variable β-defensin genomic copy number affects immune system function; in this case lung infection in a susceptible CF patient cohort. From the data we have presented, it is clear that either β-defensin genomic copy number has no effect on lung function in CF patients, or that any effect is too small for it to be detected using this cohort size. We cannot formally rule out an effect of single nucleotide polymorphisms (SNPs) within or surrounding the *DEFB4 *gene, given the apparent lack of association between copy number alleles and microsatellite alleles, and by inference any single nucleotide polymorphism (SNP). Any further study on these SNPs will have to carefully consider the copy number variation at this locus, given that a single nucleotide change could not only be allelic but differ between paralogues on the same chromosome [16].

There could be two reasons why β-defensin genomic copy number has no effect on lung function in CF patients. Firstly, unlike in cultured lymphocytes [14], there may be no correlation between *DEFB4 *copy number and gene expression in lung epithelia. Ideally a positive correlation between *DEFB4 *copy number and *DEFB4 *expression levels in non-inflamed lungs should be established, but realistically such studies are difficult to perform for both technical and ethical reasons. Even if such a direct link were proved, the lung in CF patients is characterised by a strong inflammatory response, and, given the dramatic increase in DEFB4 peptide levels in inflamed tissue [24], this could hide any differences in basal expression levels due to genomic copy number variation. Secondly, differences in *DEFB4 *expression are masked by other factors in the CF lung: for example, elastolytic cathepsins which breakdown DEFB4 and DEFB103 are present in the CF lung, but not in the healthy lung [25]. A recent report studying single nucleotide variation in the *DEFB1 *gene and lung function in CF patients has found no link, providing further evidence against beta-defensins modifying lung function in CF patients [26].

The key to further progress is to understand role of antimicrobial peptides in the inflamed lung, and exactly how they contribute to airway surface defence. A recent paper shows that DEFB4 is more than an antimicrobial peptide, and attracts neutrophils to sites of inflammation and infection [27], so it is possible that its chemoattractant role is as important in the lung as its antimicrobial role. How DEFB4 interacts with other components of the inflammatory response, and the innate and adaptive immune response, is only just beginning to be understood, and it is clear that much more work is needed before we understand these processes in lung disease.

## Methods

### Patient samples

DNA was extracted from peripheral blood from cystic fibrosis patients using the QIAamp DNA Blood Midi kit (Qiagen) according to the manufacturer's recommendations as part of a larger modifier gene study. Samples were coded and anonymised. All participants gave informed consent and the study was approved by the Royal Brompton Hospital and Harefield NHS Trust Ethics Committee.

### Clinical parameters

Clinical data were obtained from the clinical databases, patients' hospital notes and computerised microbiology reports. Forced expiratory volume in 1 second (FEV_1_) and forced vital capacity (FVC) were obtained from annual lung function laboratory records, had been corrected for sex, age and height and are expressed as percent of predicted normal values. Annual values were recorded where available from 1990–2002 and annual rate of decline was calculated for all subjects with 2 or more values after linear regression of all available data. Transcutaneous oxygen saturation with the patient breathing room air was obtained at the annual visit, and a record of the requirement for supplementary oxygen was made. Additional data included the presence of infection with *P. aeruginosa *or *B. cepacia *and most recent total white blood cell count, C-reactive protein and liver function tests.

### Multiplex amplifiable probe hybridisation (MAPH)

MAPH was carried out essentially as previously described [28,29], with fluorescent detection of amplification products by an Applied Biosystems 3100 Genetic Analyzer. A probe set including six probes mapping to the beta-defensin cluster was constructed (Table 1). The values for these six probes were normalised first for lane intensity against control subtelomeric probes in the same probe set which do not show copy number variation [20]. These ratios were then calibrated against a weighted average of two control genomic samples in the same experiment, usually N8 (3 copies) and N25 (7 copies) to get a value for the number of copies of each probe per diploid cell [14]. In two instances, two other known controls were used on duplicate samples (European Collection of Animal Cell Cultures (ECACC) samples BO0183 3 copies – AF0105 4 copies). Of the 355 samples tested, 205 were tested in duplicate. Copy number was calculated by averaging the values of each probe mapping to this region and presenting the data both as a mean value +/- 95% confidence intervals (CIs), and as a rounded integer, representing the most likely copy number. Initially all six probes were used in the calculation, but it was found that DEFB104 probe was unusually sensitive to DNA quantity and removing the values for that probe in many cases improved the confidence intervals. All data in this paper are from five probes only. The 95% CIs for each copy number reading in the full cohort ranged from 0.05 to 1.2 with a median value of 0.24.

**Table 1:** MAPH probes that detect beta-defensin copy number variation

Probe name	Hollox et al (Reference 14) probe	Genomic location	Accession number	Location in clone
DEFB106	-	DEFB106 exon 2	AF252830.5	6703–7022
DEFB104	-	DEFB104 exon 2	AF252830.5	18832–19143
DEFB4B	G	DEFB4 exon 2	NM_004942	117–237
G13705	H	SPAG11 intron 2	G13705	121–326
DEFB103	-	DEFB103 exon 1	AF252831.2	97176–97312
DEFB105	-	DEFB105 exon 3	AF202031.5	57470–57612

### Microsatellite analysis

A microsatellite (EPEV-2) in the intron of DEFB4, was amplified using the following primers (EPEV-2F 5'-GCACCAGAGACCTCATGTTTTC-3' and EPEV-2R 5'-GTAACTTACAGTTGAAAACCAC-3'), with EPEV-2F 5' fluorescently-labelled with 6-FAM. The PCR conditions were as follows: 0.2 mM each of dATP, dCTP, dGTP, dTTP, 1 mM MgCl_2_, 75 mM Tris-HCl (pH 8.8 at 25°C), 20 mM (NH_4_)_2_SO_4_, 0.01% (v/v) Tween 20 in a final volume of 10 μl, with 5 pmol of each primer, 10 ng genomic DNA, 0.5 units Taq DNA polymerase (ABgene), followed by cycling for 25 cycles at 95°C 1 minute, 60°C 1 minute 72°C 1 minute, and a final extension incubation of 72°C 20 minutes. 1.5 μl of the PCR product was mixed with 10 μl Hi-Di formamide and run on an ABI 3100 using standard conditions, with G500 ROX marker. Allelic sizes ranged from 240 bp to 272 bp, and samples were omitted from analysis if stutter peaks made the interpretation of allele peak areas or allele lengths ambiguous.

## Authors' contributions

EJH carried out the molecular laboratory work, and participated in the design of the study. JD and EJH carried out the statistical analysis, and JD together with JB participated in clinical data collection and method design. UG, EA, and JALA participated in the design of the study. All authors helped to draft the manuscript, and have read and approved the final manuscript.
